# Superconducting magnetoresistance in ferromagnet/superconductor/ferromagnet trilayers

**DOI:** 10.1038/srep13420

**Published:** 2015-08-26

**Authors:** D. Stamopoulos, E. Aristomenopoulou

**Affiliations:** 1 Institute of Nanoscience and Nanotechnology, National Center for Scientific Research ‘Demokritos’, 153 10, Aghia Paraskevi, Greece; 2 Department of Solid State Physics, National and Kapodistrian University of Athens, Zografou Panepistimioupolis, 157 84, Zografou, Greece

## Abstract

Magnetoresistance is a multifaceted effect reflecting the diverse transport mechanisms exhibited by different kinds of plain materials and hybrid nanostructures; among other, giant, colossal, and extraordinary magnetoresistance versions exist, with the notation indicative of the intensity. Here we report on the superconducting magnetoresistance observed in ferromagnet/superconductor/ferromagnet trilayers, namely Co/Nb/Co trilayers, subjected to a parallel external magnetic field equal to the coercive field. By manipulating the transverse stray dipolar fields that originate from the out-of-plane magnetic domains of the outer layers that develop at coercivity, we can suppress the supercurrent of the interlayer. We experimentally demonstrate a scaling of the magnetoresistance magnitude that we reproduce with a closed-form phenomenological formula that incorporates relevant macroscopic parameters and microscopic length scales of the superconducting and ferromagnetic structural units. The generic approach introduced here can be used to design novel cryogenic devices that completely switch the supercurrent ‘on’ and ‘off’, thus exhibiting the ultimate magnetoresistance magnitude 100% on a regular basis.

## Introduction

Magnetoresistance (MR), the change in the resistance of a specimen under the application of an external magnetic field, is a multifaceted effect that arises from the diversity of the underlying transport mechanisms exhibited by different kinds of plain materials and hybrid nanostructures. Plain materials exhibit both conventional and exotic MR effects such as anisotropic MR^1^,^2^ and colossal MR^3^,^4^. On the other hand, hybrid nanostructures of plain materials exhibit mainly intriguing MR versions, such as giant MR observed in ferromagnet/normal-metal/ferromagnet trilayers^5^,^6^ and tunnel MR observed in ferromagnet/insulator/ferromagnet ones^7^,^8^, when subjected to a parallel external magnetic field. Even stronger MR effects have been reported, the so-called extraordinary MR is recorded in normal-metal/semiconductor hybrid structures^9^,^10^, and the so-called extremely large MR is observed in layered transition-metal dichalcogenide compounds^11^. Some of these MR effects have already promoted the realization of devices that operate effectively in room-temperature and/or cryogenic-environment conditions.

Superconducting MR (SMR) is observed at cryogenic-environment conditions in ferromagnet/superconductor/ferromagnet trilayers (FM/SC/FM TLs) when subjected to a parallel external magnetic field, H_ex_^12^,^13^,^14^,^15^,^16^,^17^,^18^,^19^,^20^,^21^,^22^,^23^,^24^. Regarding the phenomenological behavior and potential for applications, SMR has much in common with some of the above mentioned MR effects. The SMR effect stems from a dipolar-field-based physical mechanism. Specifically, FM/SC/FM TLs exhibit SMR when they are driven to the coercive field, H_C_ by a parallel H_ex_^15^,^16^. The out-of-plane magnetic domains (MDs) and MDs walls (MDWs) that develop at H_C_ are accompanied by transverse stray dipolar fields that magnetostatically couple the FM outer layers through the SC interlayer^12^,^13^,^24^. Thus, the SC interlayer experiences local magnetic fields that exceed either its lower-critical field (SMR relates to vortex-motion dissipation processes) or its upper-critical field (SMR relates to the charge-dependent orbital effect)^12^,^15^. In either case, the FM/SC/FM TLs should have a predictable behavior regarding the SMR magnitude to allow the design of cryogenic devices, with the possibility to be operated as ideal supercurrent switches, exhibiting a 100% SMR magnitude under the application of a relatively low parallel H_ex_. Within this context, modeling of the SMR effect observed in FM/SC/FM TLs is needed.

In this work we employ Co/Nb/Co TLs as a model system. We present experimental data and phenomenological modeling of the SMR effect that, in these TLs, is accompanied by an intense reentrance of the upper-critical field line, H_c2_(T) close to the critical temperature^12^,^13^,^24^. We investigate many sets of Co(d_Co_)/Nb(d_Nb_)/Co(d_Co_) TLs with relatively thick Co outer layers, d_Co_ = 60 and 100 nm > 40–50 nm, so that out-of-plane MDs do emerge at H_C_, and relatively thin Nb interlayer, 15 nm < d_Nb_ < 23 nm, to enhance the SMR magnitude. We experimentally demonstrate a scaling of the SMR magnitude that is modeled by a simple closed-form phenomenological formula that incorporates the relevant macroscopic parameters and microscopic length scales of the SC and FM structural units.

### SMR effect and reentrance of the upper-critical field line, H_c2_(T)

**Figure 1:**
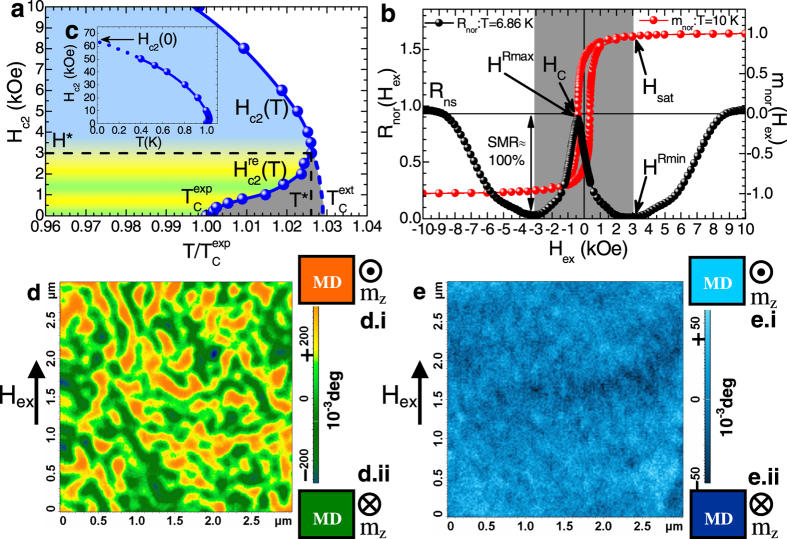
Operational phase diagram of a representative Co(100)/Nb(17)/Co(100) trilayer as constructed from transport, magnetization and magnetic force microscopy measurements. (**a**) Upper-critical field line, H_c2_(T) for a representative Co(100)/Nb(17)/Co(100) TL in the regime close to the critical temperature where the reentrance branch, H_c2_^re^(T) is evident (temperature is normalized in respect to the zero-field experimental value T_C_^exp^). The point (T^*^, H^*^) characterizes the end of reentrance, while point T_C_^ext^ is estimated from the extrapolation of the high-field part of H_c2_(T) to zero field. (**b**) Magnetoresistance curve, R_nor_(H_ex_) recorded at temperature T = 6.86 K where the ideal SMR magnitude, 100%, is observed for the specific TL, in comparison with a magnetization curve, m_nor_(H_ex_) recorded at T = 10 K (both curves are normalized for the sake of presentation). (**c**) Inset shows the entire experimentally-accessible phase diagram where the extrapolation of the high-field part of H_c2_(T) to zero temperature for the estimation of H_c2_(0) can be seen. (**d**,**e**) Representative magnetic force microscopy (MFM) images (3 × 3 μm^2^) in two-dimensional top-view form, obtained for (**d**) H_ex_ ≈ H_C_  H^*^ ≈ H_sat_ and **e** H_**e**x_ ≥ H^*^ ≈ H_sat_  H_C_, evidencing the existence and absence, respectively, of out-of-plane magnetic domains (MDs) in respect to the parallel external magnetic field, H_ex_ (notice the difference in the phase scale between the two MFM images). Color notation of both the MFM images, (**d**,**e**) and of the respective satellite cartoons, (**d.i,d.ii**,**e.i,e.ii**) that indicate the orientation of MDs magnetization (top-view form), is in accordance with that of panel (**a**).

Figure 1a–e show the operational phase diagram and representative data for a Co(100)/Nb(17)/Co(100) TL to establish the background for the subsequent modeling and discussion.

Figure 1a shows the experimentally-determined operational phase diagram in the regime close to the critical temperature where the reentrance branch, H_c2_^re^(T) of the upper-critical field line is evident. The point (T^*^, H^*^) characterizes the reentrance end. T_C_^exp^ refers to the experimentally-recorded critical temperature, while T_C_^ext^ is estimated from the extrapolation of the high-field part of H_c2_(T) to zero field. Inset, Fig. 1c, shows the entire experimentally-accessible phase diagram, where H_c2_(0) is estimated from the extrapolation of the high-field part of H_c2_(T) to zero temperature. For the extrapolation we use the relation that holds in FM/SC/FM TLs subjected to a parallel magnetic field when the coherence length, ξ(T) gets on the order of the SC thickness, d_SC_^25^


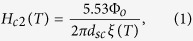


where ξ(T) = ξ(0)/(1 − T/T_C_)^1/2^ and Φ_o_ = hc/2e is the flux quantum (2.07 × 10^−7^ Gcm^2^). Accordingly, from H_c2_(0) we calculate ξ(0).

Figure 1b presents a magnetoresistance curve, R_nor_(H_ex_) recorded at temperature T = 6.86 K where the maximum SMR value (termed SMR magnitude from now on) is observed for this specific TL. The SMR value is defined from the relation ΔR/R_ns_ = (R_max_ − R_min_)/R_ns_ × 100%, where R_max_ and R_min_ refer to the maximum and minimum resistance observed upon application of H_ex_, while R_ns_ is the reference resistance of the normal state. Thus, according to this definition the ultimate SMR magnitude that, ideally, can be obtained is 100%; this refers to complete switching, ‘on’ and ‘off’, of the supercurrent of the FM/SC/FM TL^12^,^13^. This value is comparable to the giant MR observed in more complex ferromagnet/normal-metal multilayers that are deposited on rigid or flexible substrates, that is on the order of 60% and 80% for operation at room temperature and cryogenic conditions, respectively^5^,^6^,^26^,^27^. Figure 1b, also shows a magnetization curve, m_nor_(H_ex_) recorded at T = 10 K. From the comparison of the data in Fig. 1a,b a number of solid conclusions can be drawn. First, the characteristic point H^*^ where the reentrance ends (Fig. 1a) coincides with point H^Rmin^ where the resistance becomes minimum and with the saturation field, H_sat_ where the magnetization becomes maximum (Fig. 1b), that is H^*^ = H^Rmin^ = H_sat_. Second, the resistance maximum observed at the characteristic point H^Rmax^ coincides with the coercive field, H_C_ where magnetization becomes minimum (Fig. 1b), that is H^Rmax^ = H_C_. Third, the SMR effect is restricted to the regime below the saturation field, H_sat_ (Fig. 1b; gray area) where irreversible magnetization processes exist^24^.

Figure 1d,e show representative magnetic force microscopy (MFM) images in two-dimensional top-view form for the Co(100)/Nb(17)/Co(100) TL obtained for H_ex_ ≈ H_C_  H^*^ ≈ H_sat_ and H_ex_ ≥ H^*^ ≈ H_sat_  H_C_, respectively (the relevant satellite cartoons 1d.i–1d.ii and 1e.i–1e.ii show the orientation of MDs magnetization, in respect to the color scale bar). These MFM images prove the existence and absence of MDs in each case. Thus, in connection to the magnetoresistance and magnetization data, Fig. 1b, the MFM data, Fig. 1d,e, experimentally document in a direct way that the MDs are responsible for the SMR effect observed in the Co/Nb/Co TLs studied here. Also, the behavior of the upper-critical field line in the low field regime, shown in Fig. 1a, resembles the asymmetry of the respective phase boundary reported in^28^ that was driven by the uneven population of oppositely oriented MDs. Most importantly, in the Co/Nb/Co TLs studied here the SMR magnitude attains the ultimate value 100%, Fig. 1b, for reasons elucidated below.

The combined transport, magnetization and MFM measurements were performed on all sets of Co(d_Co_)/Nb(d_Nb_)/Co(d_Co_) TLs studied here to record the SC and FM macroscopic parameters and the microscopic length scales of interest (e.g. T_C_^exp^, d_SC_, d_FM_, ξ(0), D_DMs_ etc). All these data are given in Supplementary Tables 1 and 2 (for details see Supplementary Methods-Data collection of Supplementary Information). This information is crucial since its compilation on the physics grounds of the FM/SC/FM TLs will enable us to construct a phenomenological model for the magnitude of the SMR effect. To this end, first we identified which are the most important factors that affect the SMR magnitude, and then we investigated the scaling of the SMR magnitude with different combinations of the interfering macroscopic parameters and microscopic length scales of the SC interlayer and FM outer layers.

### Dependence of the SMR magnitude on the characteristics of the superconducting resistive transition

**Figure 2:**
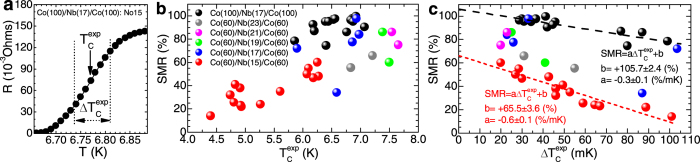
Resistive transition characteristics and dependence of the superconducting magnetoresistance magnitude. (**a**) Virgin zero-field resistive transition curve, R(T) for a representative Co(100)/Nb(17)/Co(100) TL where the critical temperature, T_C_^exp^ (50% criterion) and transition width, ΔT_C_^exp^ (20%–80% criterion) are shown. (**b**) Superconducting magnetoresistance (SMR) magnitude as function of the critical temperature, T_C_^exp^. (**c**) SMR magnitude as function of the transition width, ΔT_C_^exp^ (data points notation as in (**b**)). The dashed-black and dotted-red lines refer to linear fits for the specific sets of Co(100)/Nb(17)/Co(100) and Co(60)/Nb(15)/Co(60) TLs, respectively.

Figure 2a shows the zero-field resistive curve, R(T) for a representative Co(100)/Nb(17)/Co(100) TL to define the experimentally-determined critical temperature, T_C_^exp^ and transition width, ΔT_C_^exp^ that are basic quality indices of a SC film. Most importantly, Fig. 2b,c show the dependence of the SMR magnitude on T_C_^exp^ and ΔT_C_^exp^, respectively. Notice that the data are quite scattered when plotted against T_C_^exp^, Fig. 2b. The comparison with the data of Fig. 2c documents that the SMR magnitude depends most strongly on the transition width, ΔT_C_^exp^. Dashed-black and dotted-red lines in Fig. 2c represent linear fits of the data referring to the specific sets of Co(100)/Nb(17)/Co(100) and Co(60)/Nb(15)/Co(60) TLs, respectively. Notably, in these fits the intersection point with the vertical axis denotes the maximum SMR magnitude that can be obtained for the ideal TL of each set that exhibits infinitely sharp resistive transition (ΔT_C_^exp^ = 0). Accordingly, we clearly see that the intersection point varies among the different sets of TLs, thus it depends on the specific characteristics of the SC and FM structural units. Specifically, for the set of Co(100)/Nb(17)/Co(100) TLs the intersection point, 105.7 ± 2.4, overestimates the ideal value of the SMR magnitude, 100%, while for the set of Co(60)/Nb(15)/Co(60) TLs the intersection point, 65.5 ± 3.6, is significantly lower than 100%. A crucial task that has to be addressed in the search for a closed-form recipe to reproduce the SMR magnitude, is to find the most important SC and FM macroscopic parameters and microscopic length scales (e.g. T_C_^exp^, d_SC_, d_FM_, ξ(0), D_DMs_ etc) that affect its intensity.

### Scaling of the SMR magnitude

To address this issue, scaling plots of the SMR magnitude on the relevant SC and FM parameters and length scales can be employed. First of all we note that the SMR magnitude does not correlate with the normal state MR (nsMR in Supplementary Tables 1 and 2 of Supplementary Information), proof that the SMR effect is a purely superconducting feature. Furthermore, in Fig. 2b,c we see that the SMR magnitude does not scale neither with T_C_^exp^ nor with ΔT_C_^exp^ (the same holds for other simple factors, for instance T_C_^ext^). We examined many other factors of the SC and FM ingredients (see Supplementary Tables 1 and 2 of Supplementary Information) and found that excellent scaling is obtained when we use the ratio ΔT_C_^exp^/(T_C_^ext^ − T_C_^exp^). Intuitively, this factor is expected to play dominant role since the numerator refers to the width of the resistive transition, Fig. 2a, while the denominator refers to the shift of the critical temperature under the application of the MDs, Fig. 1a. Thus, when (T_C_^ext^ − T_C_^exp^) exceeds ΔT_C_^exp^ it is expected that the SMR magnitude should attain its maximum value, 100%.

**Figure 3:**
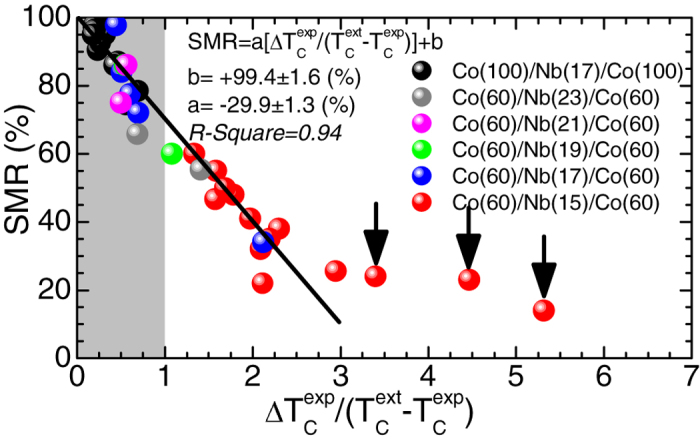
Scaling of the superconducting magnetoresistance magnitude. Scaling of the superconducting magnetoresistance (SMR) magnitude with the ratio ΔT_C_^exp^/(T_C_^ext^ − T_C_^exp^). The solid-black line represents linear fit of the SMR magnitude for the complete data referring to all sets of Co(d_Co_)/Nb(d_Nb_)/Co(d_Co_) TLs, with the exception of three data points (Co(60)/Nb(15)/Co(60) TLs) marked with vertical arrows. Gray surveys the area where the reentrance of branch H_c2_^re^(T) exceeds the width of the resistive transition, ΔT_C_^exp^ ((T_C_^ext^ − T_C_^exp^) > ΔT_C_^exp^) so that ideal SMR magnitude, 100%, is established.

The scaling of the SMR magnitude on the factor ΔT_C_^exp^/(T_C_^ext^ − T_C_^exp^) is shown in Fig. 3. The solid-black line represents a linear fit of the complete data referring to all sets of Co(d_Co_)/Nb(d_Nb_)/Co(d_Co_) TLs, with the exception of three specific data points of Co(60)/Nb(15)/Co(60) TLs marked with vertical arrows. Notably, the area ΔT_C_^exp^/(T_C_^ext^ − T_C_^exp^) > 3 possibly refers to a second mechanism with a distinct scaling behavior. However, due to lack of data in this regime we do not discuss this option. Accordingly, we focus on the area ΔT_C_^exp^/(T_C_^ext^ − T_C_^exp^) < 3. By using


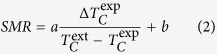


we get an impressive outcome; the intersection point with the vertical axis reads b = 99.4 ± 1.6% that within standard uncertainty is identical to the maximum value 100% expected for the ideal case of complete switching, ‘on’ and ‘off’, of the supercurrent of the FM/SC/FM TL^12^,^13^. Obviously, the slope α = −29.9 ± 1.3% depends on the criterion used to define ΔT_C_^exp^ (20%–80% in our case). This scaling plot provides important information, since once we determine the factors that govern its behavior we will be able to predict the value of the SMR magnitude in every FM/SC/FM TL, providing the technical foundation for the design of relevant cryogenic devices. To this end, a direct comparison of the data shown in Figs 2c and 3 reveals that the introduction of the factor (T_C_^ext^ − T_C_^exp^) in the denominator reconfigures the experimental data of Fig. 2c so that excellent scaling is realized in Fig. 3. *Thus, the factor (T*_*C*_^*ext*^* − T*_*C*_^*exp*^*) renormalizes the SMR magnitude on the basis of the different macroscopic parameters and microscopic length scales (e.g. T*_*C*_^*exp*^*, d*_*SC*_*, d*_*FM*_*, ξ(0), D*_*DMs*_*etc) of the SC and FM structural units of the FM/SC/FM TLs.* Consequently, the construction of a model that will enable us to estimate the factor (T_C_^ext^ − T_C_^exp^) on the basis of these quantities is of paramount importance and is discussed in the next section.

### Modeling the suppressed critical temperature T_C_^exp^ with respect to T_C_^ext^

In earlier works^12^,^13^,^24^ we have seen that the reentrance branch, H_c2_^re^(T) in the low-field regime, close to the critical temperature, can be ascribed to the suppression of superconductivity by the transverse stray dipolar fields that emerge at the interiors of out-of-plane MDs as the coercive field, H_C_, is approached. Nevertheless, we have to keep in mind that MDWs assist the nucleation of superconductivity^29^,^30^. Thus, the intrinsic critical temperature, that originally should be T_C_^ext^, is suppressed to T_C_^exp^ (Fig. 1a) under the action of the main body of MDs when MDWs are excluded; the suppression of T_C_^ext^ to T_C_^exp^ is driven only by the net width D_MDs_-D_MDWs_, where D_MDs_ and D_MDWs_ is the width of MDs and MDWs, respectively. On this basis, we propose that the experimentally-determined suppressed critical temperature, T_C_^exp^ (Fig. 1a) can be estimated from the condition ξ(T_C_^exp^) = D_MDs_ − D_MDWs_, that is T_C_^exp^ denotes the temperature where the coherence length, ξ(T) equals to the net width of MDs, D_MDs_-D_MDWs_. We propose this condition since, when it holds, the superconducting nucleus can no longer be benefited by the selective localization above MDWs, since it necessarily extends over entire MDs, thus is forced to experience the hostile transverse stray dipolar fields. By using relation ξ(T) = ξ(0)/(1 − T/T_C_)^1/2^, in which for T_C_ we insert the intrinsic critical temperature, T_C_^ext^ (Fig. 1a), after simple algebra we obtain the equation


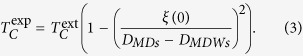


Equation 3 shows that the experimentally-determined critical temperature, T_C_^exp^ depends on the ratio ξ(0)/(D_MDs_-D_MDWs_); for fixed ξ(0), the wider/narrower the MDs, the weaker/stronger the suppression, while for fixed D_MDs_-D_MDWs_, the lower/higher the ξ(0) the weaker/stronger the suppression.

Equation 3 refers to purely experimental quantities that are given in Supplementary Tables 1 and 2 of Supplementary Information. Thus, it can be used to test the self-consistency of our approach to estimate T_C_^exp^ with respect to the T_C_^ext^. For instance, equation 3 performs excellently when we use it to estimate the critical temperature shift (T_C_^ext^ − T_C_^exp^) and compare it with the purely experimental data for the Co(100)/Nb(17)/Co(100) TLs. The mean value <T_C_^ext^ − T_C_^exp^> estimated using equation 3 in combination with the data of Supplementary Table 1 of Supplementary Information yields <T_C_^ext^ − T_C_^exp^> = 0.164 ± 0.030 K, while the purely experimental value directly calculated from the data of Supplementary Table 1 of Supplementary Information gives <T_C_^ext^ − T_C_^exp^> = 0.167 ± 0.020 K. Notably, if we ignore the length scale D_DMWs_ in the denominator of equation 3 we seriously underestimate the shift value <T_C_^ext^ − T_C_^exp^> = 0.118 ± 0.021 K.

### Modeling the SMR magnitude

Once the proposed model-based estimation of T_C_^exp^ has been experimentally validated we can now use equation 3 to calculate the factor (T_C_^ext^ − T_C_^exp^) and substitute it for the denominator of equation 2 (linear fit of Fig. 3). With simple algebra we obtain for the SMR magnitude


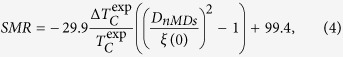


where ‘nMDs’ stands for ‘net MDs’, with D_nMDs_ = D_MDs_-D_MDWs_ the net width of MDs.

Though simple, phenomenological equation 4 contains much information on the underlying physics of the SMR effect observed in FM/SC/FM TLs and takes into account a number of the relevant macroscopic parameters and microscopic length scales (T_C_^exp^, ΔT_C_^exp^, D_MDs_, D_MDWs_ and ξ(0)). Below we proceed with the quantitative evaluation of equation 4 success in modeling the experimental data.

### Comparison of experimental data with the phenomenological model of the SMR magnitude

**Figure 4:**
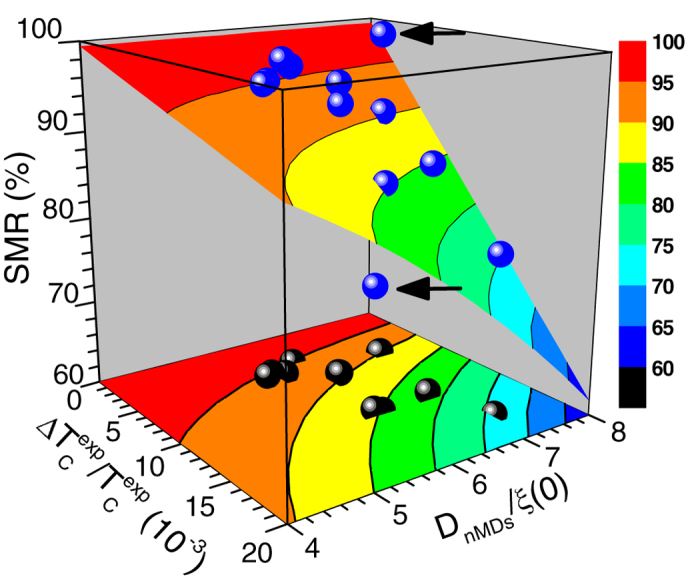
Simulation of the superconducting magnetoresistance magnitude. Simulation, based on equation 4, of the superconducting magnetoresistance (SMR) magnitude on factors ΔT_C_^exp^/T_C_^exp^ and D_nMDs_/ξ(0). Experimental data points on SMR magnitude (blue spheres; raw data, black spheres; projection on the base plane) refer to Co(100)/Nb(17)/Co(100) TLs (see Supplementary Table 1 of Supplementary Information). Horizontal arrows mark two data points that do not fit the simulation surface.

Figure 4 shows a representative simulation of the SMR magnitude, based on equation 4, on factors ΔT_C_^exp^/T_C_^exp^ and D_nMDs_/ξ(0) for the range of values met in the Co(d_Co_)/Nb(d_Nb_)/Co(d_Co_) TLs studied here (see Supplementary Tables 1 and 2 of Supplementary Information). In this simulation we have included experimental data for the set of Co(100)/Nb(17)/Co(100) TLs (see Supplementary Table 1 of Supplementary Information). From Fig. 4 we clearly see that the experimental data points (blue circles) fit well with the simulation surface (except for the ones marked with black arrows). As expected, upon decrease of factors ΔT_C_^exp^/T_C_^exp^ and D_nMDs_/ξ(0) higher SMR values can be clearly seen by their projection (black circles) on the color-scaled base plane.

From the successful comparison of equation 4 with the experimental data we conclude that equation 4 has notable predictive worth. The generic approach introduced here guarantees that equation 4 can be used to reliably design FM/SC/FM TLs that exhibit the desired SMR magnitude with the opportunity to attain even the ultimate SMR magnitude, 100%. Such FM/SC/FM TLs can act as ideal supercurrent switches that operate between two distinct states, ‘on’ and ‘off’. The wealth of experience that already exists on the design and realization of FM nanostructures that exhibit out-of-plane MDs at low coercive fields, H_C_ guarantees that relevant FM/SC/FM TLs can find wide application as sensor and storage cryogenic devices which must operate at low values of H_ex_.

## Conclusions

In summary, we have demonstrated that in Co/Nb/Co TLs with adequately thick Co outer layers and sufficiently thin Nb interlayer, an intense SMR effect emerges under the application of a parallel external magnetic field, H_ex_ equal to the coercive field, H_C_. By manipulating the transverse stray dipolar fields that originate from the out-of-plane MDs and MDWs that develop at H_C_ we can suppress the supercurrent of the Nb interlayer. We modeled the experimental data from many sets of Co(d_Co_)/Nb(d_Nb_)/Co(d_Co_) TLs with systematic variation of d_Co_ and d_Nb_ and experimentally demonstrated a scaling of the SMR magnitude. We derived a simple closed-form phenomenological formula that accurately reproduces the SMR magnitude by incorporating relevant macroscopic parameters and microscopic length scales of the SC and FM structural units. Since the outcome of this work is of generic nature, we expect that it can be employed to predict the performance of every kind of FM/SC/FM TLs and even design TLs exhibiting the ultimate SMR magnitude, 100%, at low H_ex_, on a regular basis.

## Methods

### Sample preparation

Samples were prepared on Si [100] substrates using high-purity Nb and Co targets (typically 99.9%) with a magnetron sputtering unit after adequate pumping, pre-sputtering and cryo-trapping to achieve an ultimate base pressure at the low end of 10^−8^ Torr. Details can be found in^12^.

### Magnetization measurements

Magnetization measurements were obtained at T = 10 K > T_C_^exp^, T_C_^ext^ with a commercial SQUID magnetometer. In all magnetization experiments the external magnetic field, H_ex_ was parallel to the sample surface. For details on data collection see Supplementary Information.

### Transport measurements

Transport properties were investigated, using the SQUID unit, around the experimentally-determined critical temperature T_C_^exp^ in the standard four-point in-line configuration. The applied transport current was always normal to the external magnetic field, H_ex_. In all transport experiments the external magnetic field, H_ex_ was parallel to the sample surface. Details can be found in^12^. For details on data collection see Supplementary Information.

### Magnetic force microscopy measurements

Magnetic force microscopy (MFM) data were acquired at room temperature T = 300 K with a scanning probe microscope using cantilevers with the nominal parameters of tip radius < 50–60 nm, spring constant = 2.8–3.0 Nm^−1^ and resonance frequency = 75 kHz. The MFM data were acquired both at the coercive field, H_C_ and at the saturation field, H_sat_. In all MFM experiments the external magnetic field, H_ex_ was parallel to the samples. Details can be found in^12^. For details on data collection see Supplementary Information.

## Additional Information

**How to cite this article**: Stamopoulos, D. and Aristomenopoulou, E. Superconducting magnetoresistance in ferromagnet/superconductor/ferromagnet trilayers. *Sci. Rep.* **5**, 13420; doi: 10.1038/srep13420 (2015).

## Supplementary Material

Supplementary Information
