# Erratum: Superconducting magnetoresistance in ferromagnet/superconductor/ferromagnet trilayers

**DOI:** 10.1038/srep15094

**Published:** 2015-10-30

**Authors:** D. Stamopoulos, E. Aristomenopoulou

Scientific Reports 5: Article number: 1342010.1038/srep13420; published online: 08262015; updated: 10302015

In the HTML version of this Article, the legend of Figure 1 contains typographical errors.

“(**d**) H_ex_ ≈ H_C _H^*^ ≈ H_sat_ and **e** H_**e**x_ ≥ H^*^ ≈ H_sat _H_C_”

should read:

“(**d**) H_ex_ ≈ H_C _≪H^*^ ≈ H_sat_ and (**e**) H_**e**x _≥ H^*^ ≈ H_sat _≫ H_C_”

