# Supplementary material for: Superconducting magnetoresistance in ferromagnet/superconductor/ferromagnet trilayers
Source: Sci Rep. 2015 Aug 26;5:13420. doi: 10.1038/srep13420 (PMC4549620; doi:10.1038/srep13420)
Supplement: Supplementary Information [file srep13420-s1.doc]

**Supplementary Information for manuscript entitled:**

**Superconducting magnetoresistance in ferromagnet/superconductor/ferromagnet trilayers**

D. Stamopoulos^1,2^,* and E. Aristomenopoulou^1^

1.Institute of Nanoscience and Nanotechnology, National Center for Scientific Research 'Demokritos', 153 10, Aghia Paraskevi, Greece

2. Department of Solid State Physics, National and Kapodistrian University of Athens, Zografou Panepistimioupolis, 157 84, Zografou, Greece

***** Corresponding author: [d.stamopoulos@inn.demokritos.gr](mailto:d.stamopoulos@inn.demokritos.gr) and [densta@phys.uoa.gr](mailto:densta@phys.uoa.gr)

**Supplementary Table 1**

Co(100nm)/Nb(17nm)/Co(100nm)

| **Co(100nm)/Nb(17nm)/Co(100nm)** | | | | | | | | | | | | | | | | | |
| --- | --- | --- | --- | --- | --- | --- | --- | --- | --- | --- | --- | --- | --- | --- | --- | --- | --- |
|  | **SC parameters and length scales** | | | | | | | | | **FM parameters and length scales** | | | |  | | | |
| **#** | **d_sc_**  **(nm)** | **T_c_[100%]**  **(K)** | **ΔΤ_c_[20%-80%]**  **(mK)** | **T_c_^exp^[50%]**  **(K)** | **T***  **(K)** | **T_c_^ext^[50%]**  **(K)** | **H_c2_(0)**  **kOe** | **ξ(0)**  **(nm)** | **H***  **(kOe)** | **d_FM_**  **(nm)** | **H_sat_**  **(kOe)** | **D_MDS_**  **(nm)** | **D_MDWs_**  **(nm)** | **nsMR**  **(%)** | **SMR**  **(%)** | **T*-T_c_^exp^**  **(mK)** | **T_c_^ext^-T_c_^exp^**  **(mK)** |
| **Nο1** | 17 | 6.650 | 34.0 | 6.527 | 6.667 | 6.695 | 67.240 | 15.9 | 4.047 | 100 | 4.033 | - | - | 0.4 | 96.8 | 140 | 168 |
| **Nο2** | 17 | 6.950 | 36.6 | 6.859 | 7.002 | 7.048 | 67.235 | 15.9 | 3.927 | 100 | 3.900 | 113.8 | 19.6 | 0.5 | 97.6 | 143 | 189 |
| **Nο3** | 17 | 7.150 | 42.0 | 7.068 | 7.218 | 7.246 | 70.509 | 15.2 | 3.310 | 100 | 3.247 | 129.5 | 18.9 | 0.2 | 90.5 | 150 | 178 |
| **Nο4** | 17 | 7.020 | 43.6 | 6.921 | 7.076 | 7.103 | 69.489 | 15.4 | 3.982 | 100 | 3.959 | 127.2 | 13.9 | 0.3 | 99.6 | 155 | 182 |
| **Nο5** | 17 | 6.660 | 30.0 | 6.558 | 6.690 | 6.716 | 65.539 | 16.4 | 3.961 | 100 | 3.990 | - | - | 0.5 | 97.5 | 132 | 158 |
| **Nο6** | 17 | 6.500 | 45.7 | 6.392 | 6.515 | 6.536 | 64.766 | 16.6 | 4.020 | 100 | 3.974 | 126.9 | 19.8 | 0.6 | 95.0 | 123 | 144 |
| **Nο7** | 17 | - | - | - | - | - | - | - | - | 100 | 4.151 | 116.4 | 19.2 | - | 100.0 | - | - |
| **Nο8** | 17 | 6.550 | 31.0 | 6.445 | 6.568 | 6.602 | 61.040 | 17.6 | 4.018 | 100 | 3.964 | 120.2 | 18.2 | 0.3 | 95.2 | 123 | 157 |
| **Nο9** | 17 | 6.330 | 44.6 | 6.176 | 6.319 | 6.344 | 69.274 | 15.5 | 4.016 | 100 | 3.935 | 122.2 | 22.0 | 0.2 | 92.6 | 143 | 168 |
| **Nο10** | 17 | 6.350 | 77.0 | 6.197 | 6.336 | 6.362 | 70.360 | 15.2 | 3.953 | 100 | 3.994 | 126.7 | 20.9 | 0.5 | 87.0 | 139 | 165 |
| **Nο11** | 17 | 6.020 | 99.0 | 5.859 | 5.983 | 6.002 | 67.340 | 15.9 | 3.534 | 100 | 3.468 | 129.2 | 15.2 | 0.4 | 78.5 | 124 | 143 |
| **Nο12** | 17 | 6.570 | 81.5 | 6.403 | 6.519 | 6.550 | 64.701 | 16.6 | 4.006 | 100 | 4.018 | 122.0 | 19.7 | 0.3 | 74.5 | 116 | 147 |
| **Nο13** | 17 | 6.890 | 28.7 | 6.832 | 6.955 | 6.973 | 62.495 | 17.2 | 2.994 | 100 | 2.976 | 127.1 | 20.8 | 0.2 | 96.4 | 123 | 141 |
| **Nο14** | 17 | 6.970 | 34.4 | 6.872 | 7.041 | 7.065 | 64.596 | 16.6 | 3.479 | 100 | 3.315 | 111.6 | 16.8 | 0.2 | 95.0 | 169 | 193 |
| **Nο15** | 17 | 6.910 | 87.0 | 6.759 | 6.935 | 6.965 | 64.195 | 16.7 | 3.495 | 100 | 3.468 | 120.8 | 16.8 | 0.3 | 86.0 | 176 | 206 |

**Supplementary Table 2**

| **Co(60nm)/Nb(15nm)/Co(60nm)** | | | | | | | | | | | | | | | | | |
| --- | --- | --- | --- | --- | --- | --- | --- | --- | --- | --- | --- | --- | --- | --- | --- | --- | --- |
|  | **SC parameters and length scales** | | | | | | | | | | | **FM parameters and length scales** | |  | | | |
| **#** | **d_sc_**  **(nm)** | | **T_c_[100%]**  **(K)** | **ΔΤ_c_[20%-80%]**  **(mK)** | **T_c_^exp^[50%]**  **(K)** | **T***  **(K)** | **T_c_^ext^[50%]**  **(K)** | **H_c2_(0)**  **kOe** | **ξ(0)**  **(nm)** | **H***  **(kOe)** | | **d_FM_**  **(nm)** | **H_sat_**  **(kOe)** | **nsMR**  **(%)** | **SMR**  **(%)** | **T*-T_c_^exp^**  **(mK)** | **T_c_^ext^-T_c_^exp^**  **(mK)** |
| **No1** | 15 | | 5.000 | 46.0 | 4.913 | 4.932 | 4.933 | 78.349 | 15.5 | 1.415 | | 60 | 1.413 | 0.2 | 38.0 | 19 | 20 |
| **No2** | 15 | | 5.070 | 88.8 | 4.933 | 4.973 | 4.975 | 77.792 | 15.6 | 0.997 | | 60 | 1.016 | 0.6 | 22.0 | 40 | 42 |
| **No3** | 15 | | 4.800 | 46.0 | 4.740 | 4.760 | 4.762 | 77.541 | 15.7 | 1.205 | | 60 | 1.208 | 0.4 | 32.0 | 20 | 22 |
| **No4** | 15 | | 5.370 | 64.6 | 5.265 | 5.283 | 5.284 | 75.518 | 16.1 | 1.017 | | 60 | 1.004 | 0.4 | 24.0 | 18 | 19 |
| **No5** | 15 | | 5.050 | 67.0 | 4.921 | 4.935 | 4.936 | 70.586 | 17.2 | 0.592 | | 60 | 0.566 | 0.6 | 23.0 | 14 | 15 |
| **No6** | 15 | | 4.530 | 101.0 | 4.399 | 4.415 | 4.418 | 67.268 | 18.1 | 0.506 | | 60 | 0.501 | 0.8 | 14.0 | 16 | 19 |
| **No7** | 15 | | 4.870 | 59.0 | 4.802 | 4.821 | 4.822 | 73.163 | 16.6 | 0.810 | | 60 | 0.792 | 0.4 | 25.6 | 19 | 20 |
| **No8** | 15 | | 6.340 | 42.8 | 6.273 | 6.295 | 6.297 | 78.399 | 15.5 | 0.699 | | 60 | 0.71 | 0.2 | 48.0 | 22 | 24 |
| **No9** | 15 | | 6.280 | 20.0 | 6.252 | 6.265 | 6.267 | 78.013 | 15.6 | 0.902 | | 60 | 0.899 | 1.2 | 60.0 | 13 | 15 |
| **No10** | 15 | | 5.670 | 52.7 | 5.608 | 5.630 | 5.632 | 75.312 | 16.1 | 0.998 | | 60 | 0.999 | 0.6 | 35.0 | 22 | 24 |
| **No11** | 15 | | 4.900 | 49.4 | 4.826 | 4.850 | 4.851 | 71.220 | 17.1 | 0.806 | | 60 | 0.814 | 0.3 | 41.0 | 24 | 25 |
| **No12** | 15 | | 6.130 | 28.5 | 6.096 | 6.113 | 6.114 | 81.101 | 14.9 | 0.814 | | 60 | 0.948 | 0.5 | 55.0 | 17 | 18 |
| **No13** | 15 | | 6.220 | 30.0 | 6.183 | 6.201 | 6.202 | 79.348 | 15.3 | 0.907 | | 60 | 0.901 | 1.0 | 46.7 | 18 | 19 |
| **No14** | 15 | | 6.100 | 27.0 | 6.068 | 6.082 | 6.084 | 79.783 | 15.2 | 1.205 | | 60 | 1.201 | 0.2 | 50.0 | 14 | 16 |
| **Co(60nm)/Nb(17nm)/Co(60nm)** | | | | | | | | | | | | | | | | | |
| **No1** | 17 | 6.975 | | 33.0 | 6.930 | 6.988 | 7.004 | 67.783 | 15.8 | | 2.430 | 60 | 2.247 | 0.5 | 97.7 | 58 | 74 |
| **No2** | 17 | 6.700 | | 87.0 | 6.587 | 6.621 | 6.628 | 64.236 | 16.7 | | 2.012 | 60 | 1.946 | 0.6 | 34.0 | 34 | 41 |
| **Ne/ns tr.** | 17 | 6.070 | | 103.6 | 5.900 | 6.038 | 6.049 | 83.179 | 12.9 | | 3.040 | 60 | 2.951 | 0.3 | 72.0 | 138 | 149 |
| **NSS 1** | 17 | 6.900 | | 26.0 | 6.861 | 6.894 | 6.904 | 68.361 | 15.7 | | 2.035 | 60 | 1.998 | 0.5 | 77.5 | 33 | 43 |
| **NSS 2** | 17 | 7.016 | | 23.0 | 6.975 | 7.015 | 7.020 | 68.488 | 15.7 | | 1.849 | 60 | 1.998 | 0.5 | 84.0 | 40 | 45 |
| **Co(60nm)/Nb(19nm)/Co(60nm)** | | | | | | | | | | | | | | | | | |
| **No1** | 19 | 7.420 | | 25.0 | 7.383 | 7.423 | 7.429 | 63.030 | 15.2 | | 1.524 | 60 | 1.511 | 0.9 | 86.0 | 40 | 46 |
| **No2** | 19 | 7.560 | | 41.0 | 7.497 | 7.532 | 7.535 | 61.829 | 15.5 | | 1.636 | 60 | 1.573 | 1.4 | 60.0 | 35 | 38 |
| **Co(60nm)/Nb(21nm)/Co(60nm)** | | | | | | | | | | | | | | | | | |
| **No1** | 21 | 7.580 | | 23.0 | 7.545 | 7.579 | 7.586 | 56.581 | 15.3 | | 1.608 | 60 | 1.603 | 1.3 | 86.0 | 34 | 41 |
| **No2** | 21 | 7.690 | | 20.0 | 7.655 | 7.685 | 7.695 | 54.208 | 16.0 | | 1.625 | 60 | 1.561 | 0.8 | 75.0 | 30 | 40 |
| **Co(60nm)/Nb(23nm)/Co(60nm)** | | | | | | | | | | | | | | | | | |
| **No1** | 23 | 6.950 | | 55.0 | 6.833 | 6.862 | 6.872 | 45.871 | 17.3 | | 1.425 | 60 | 1.439 | 0.8 | 55.4 | 29 | 39 |
| **No2** | 23 | 7.255 | | 31.0 | 7.208 | 7.241 | 7.253 | 47.465 | 16.7 | | 1.400 | 60 | 1.389 | 0.5 | 65.8 | 33 | 45 |

**Co(60nm)/Nb(d_Nb_)/Co(60nm)**

**Supplementary Methods-Data collection**

The experimental data, given in Supplementary Tables 1 and 2, was obtained following the data collection methods presented below.

**Magnetization experiments-Data collection.** From the magnetization experiments we obtain the coercive field, H_C_ and saturation field, H_sat_ (Fig. 1b of the paper).

**Transport experiments-Data collection.** The experimentally-determined critical temperature, T_C_^exp^ (50% criterion) and the respective transition width, ΔT_C_^exp^ (20%-80% criterion) are directly recorded from the virgin zero-field R(T) curves (Fig. 2a of the paper). The magnitude of SMR, ΔR/R_ns_=((R_max_-R_min_)/R_ns_)x100%, where R_ns_ is the normal-state resistance (Fig. 1b of the paper), and the reentrance branch, H_c2_^re^(T) are recorded from the set of isothermal R(H) curves obtained in the vicinity of T_C_^exp^ (Fig. 1a of the paper). The complete set of isothermal R(H) curves across the entire temperature-magnetic field regime defines the upper-critical field line, H_c2_(T) (Fig. 1a of the paper), which we reproduce by using equation (1) given in the paper. Accordingly, by using this equation we extrapolate to both zero-field to estimate T_C_^ext^ (Fig. 1a of the paper), and to zero-temperature to estimate H_c2_(0) (Fig. 1c of the paper), from which we calculate ξ(0). From the constructed phase diagram we obtain the characteristic point (T*,H*) (Fig. 1a of the paper). Finally, from the complete set of isothermal R(H) curves obtained across the resistive transition we record the one that exhibes the maximum SMR value, that we call SMR magnitude.

**Magnetic force microscopy experiments-Data collection.** From the magnetic force microscopy data obtained at H_C_ (Fig. 1d of the paper) we estimated the width of magnetic domains (MDs), D_MDs_ and width of MDs walls (MDWs), D_MDWs_ (details on the methodology can be found in Ref. 24 of the paper).
